# Antiviral, Antibacterial, Antifungal, and Anticancer Activity of Plant Materials Derived from *Cymbopogon citratus* (DC.) Stapf Species

**DOI:** 10.3390/ph17060705

**Published:** 2024-05-29

**Authors:** Anna Kiełtyka-Dadasiewicz, Javier Esteban, Agata Jabłońska-Trypuć

**Affiliations:** 1 Department of Plant Production Technology and Commodities Science, University of Life Sciences in Lublin, 20-950 Lublin, Poland; anna.kieltyka-dadasiewicz@up.lublin.pl; 2 Garden of Cosmetic Plants and Raw Materials, Research and Science Innovation Centre, 20-819 Lublin, Poland; 3 Instituto de Bioingeniería, Universidad Miguel Hernández de Elche, 03202 Elche, Spain; jesteban@umh.es; 4 Division of Chemistry, Biology and Biotechnology, Faculty of Civil Engineering and Environmental Sciences, Bialystok University of Technology, 15-351 Białystok, Poland

**Keywords:** *Cymbopogon citratus*, cancer, viruses, bacteria, fungi, essential oil, extract

## Abstract

The importance of natural plant materials in modern medicine is considerable, and raw materials with antiviral, antibacterial, antifungal, and anticancer properties are still sought because of microbe resistance and difficulties in anticancer therapy. This review focuses on the lemongrass *Cymbopogon citratus* (DC.) Stapf. and on the lemongrass oil properties and applications. Multiple applications of this plant were described in different latitudes and cultures, including cases of digestive disorders and anti-inflammatory, antipyretic, diaphoretic, stimulating, and antispasmodic conditions. Data from the literature on the composition of essential oil and extracts from *C. citratus* were analyzed, and the results of research on the antifungal, antibacterial, and antiviral effects were quoted. Essential oil inhibits the growth of fungi (*Aspergillus niger*, *A. fumigatus*, *Candida* spp.) and has an antibacterial effect (*Staphylococcus aureus*, *Bacillus subtilis*, *Pseudomonas aeruginosa*). It also shows antiviral activity and deters insects. Lemongrass contains active substances with potential anticancer effects. This plant has apoptosis-stimulating properties, mainly through the activity of apigenin, which is the main active flavonoid in this plant. This active substance helps inhibit cell proliferation by stopping the cell cycle and directing cancer cells toward apoptosis.

## 1. Introduction

**Figure 1:**
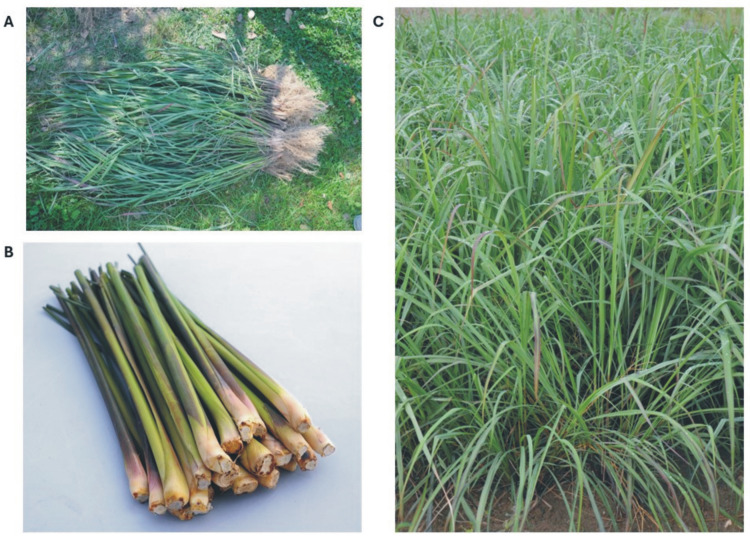
Depiction of *Cymbopogon citratus* cultivated on experimental plots of the Garden of Cosmetic Plants and Raw Materials (Research and Science Innovation Centre): (**A**) Collection of whole plants; (**B**) Cleaned shoots; (**C**) Plants before harvesting.

According to WHO data from the 2007 report, 4 billion people in the world, representing 80% of the human population, use herbal preparations to treat common health conditions [1]. Medicines and supplements of plant origin are a major element in various types of traditional medicine such as Ayurveda, homeopathy, traditional oriental medicine, and Indian medicine. It is also estimated that among plant-derived medical devices, approximately 74% are used in modern medicine in a way that correlates directly with their traditional use in folk medicine [2,3,4,5,6]. About one-fourth of annually prescribed drugs and about 7000 different medical devices contain ingredients of plant origin or their derivatives, with a market value of about USD 40 billion. Nearly 33% of medicines produced in highly developed countries are preparations obtained from plants [3,4,5,6]. One such commonly used medicinal plant is lemongrass (*Cymbopogon citratus* (DC.) Stapf) (Figure 1).

*Cymbopogon citratus* (DC.) Stapf, previously described as *Andropogon citratus* by De Candolle and reclassified by Otto Stapf, belongs to the *Poaceae* family, which includes approximately 500 genera and 8000 species of plants, commonly called grasses. The *Cymbopogon* genus includes 30 species of grasses native to Old World countries. The name “*Cymbopogon*” comes from a combination of the Greek words “kymbe”—boat and “pogon”—beard and refers to the arrangement of flower spikes. The part “*citratus*” refers to the Latin term meaning lemon-scented leaves [7]. Shah et al. [8] provide common names for Cymbopogon citratus, which is used in various regions of the world—Brazil: capim-cidrao, capim-santo, Egypt: lemon grass, Great Britain: lemongrass, citronella, squinant, Ethiopia: tej-sar, India: sera, verveine, Indonesia: sereh, Italy: cimbopogone, Malaysia: sakumau, Mexico: zacate limon, Germany: zitronengras, Sweden: citrongräss, Thailand: ta-khrai, Turkey: limon out, and the USA: citronella [7,8,9].

*C. citratus* comes from the southwest part of Asia and now grows in all tropical regions of the world and on savannahs. It is a perennial plant that grows in tall, 2-m clumps with short roots. It has long leaves up to 1 m long and 5–15 mm wide. It has an inflorescence composed of spikes arranged on the top of the blade [10]. It is also possible to grow this species in a one-year system in temperate climate conditions [11].

Although numerous literature data present *Cymbopogon citratus* as a plant source of biologically active ingredients with a broad spectrum of action, this review aims to critically evaluate the pharmacological activity not only of the essential oil as the most popular product obtained from this plant but also of other therapeutic products. A new approach in this review is to combine and summarize information regarding the origin of *Cymbopogon citratus*, its use, the chemical composition of products obtained from it, and the antibacterial, antiviral, antifungal, and anticancer effects of this plant’s raw material. Although there are many varieties and species of the genus *Cymbopogon*, this work focuses on the one selected species and takes into account its chemical composition, the presence of biologically active substances, and their health effects based on clinical evidence. The work will present the mechanisms of anticancer effects of various products obtained from *C. citratus* and active ingredients obtained from this plant. Finally, we will indicate future research directions that should be undertaken to continue the search for active compounds with anticancer properties and to prioritize further progress in the use of *C. citratus* as a raw material for medicinal use. To achieve the above objectives, a review of the literature from the last 20 years was performed, with particular emphasis on the latest research.

## 2. *Cymbopogon citratus* Application

**Figure 2:**
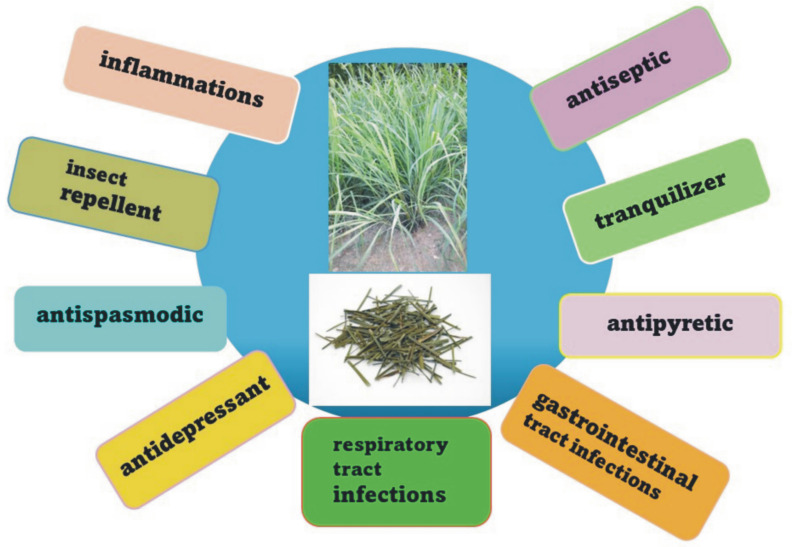
Traditional applications of the *Cymbopogon citratus*.

Infusions and decoctions prepared from fresh or dried lemongrass leaves are popular and often used almost all over the world in order to relieve various ailments. Chemical compounds extracted from *C. citratus* have an equally wide range of applications, and essential oil obtained from this plant is particularly common. In India, lemongrass is used for gastrointestinal ailments, while in China, it is used as a component of antidepressant mixtures. In the Malay Peninsula, it is commonly used to treat flu, fever, pneumonia, and gastrointestinal problems and as a diaphoretic drug. In Nigeria, it is used as an antipyretic and for its stimulating and antispasmodic properties, while in Indonesia, the plant is recommended as an ingredient that aids in digestion, promotes diuresis and sweating, and regulates the menstrual cycle. In Africa and Asia, its antitussive, antiseptic, diaphoretic, and antirheumatic properties are known, and it is used to treat lower back pain, sprains, and hemoptysis (Figure 2) [12,13,14,15,16,17].

Lemongrass is a commonly used medicine in traditional folk medicine in Cuba and many other countries in the Caribbean region. In addition to the above-mentioned properties, its analgesic and anti-inflammatory effects are also widely known and important. In the Trinidad and Tobago region, it is a popular herb used to fight diabetes. In traditional Suriname medicine, it is used against cough, wounds, asthma, and bladder diseases and as a diaphoretic and headache reliever. It is also applied as a repellent and carminative. In Brazil, it is a very common herbal medicine recommended for many ailments, including as a tonic, digestive aid, antitussive, anti-cold, analgesic, treatment of heart disease, antipyretic, anti-inflammatory in urinary tract diseases, diuretic, antispasmodic, diaphoretic, and antiallergic. There is also evidence of lemongrass being used as a mild sedative [16,18,19,20]. In addition to medicinal uses, lemongrass—most often as an essential oil obtained from it—is widely used in the food, perfume, and cosmetics industries. The oil is part of some oils applied for massage and aromatherapy. Lemongrass is also an important ingredient in oriental cuisine. Very diluted lemongrass oils are used in the food industry to flavor food products and drinks. However, in undiluted form, they may be toxic or even fatal if a large dose is taken orally. Due to the high content of citronellal, the oil is used as a repellent [11,21,22,23,24,25,26,27,28,29,30,31].

## 3. Chemical Composition of *Cymbopogon citratus* Essential Oil and Extracts

Among the many species and varieties of the genus *Cymbopogon*, the species *C. citratus*, in particular, is cultivated for its essential oil, which is used in the cosmetics, perfumery, and food industries. Its wide range of applications results from the high citral content (70–80%) [32,33,34,35,36,37,38,39]. The total amount of essential oil obtained from the leaves ranges from 0.28 to 1.4%. The maximum reported isolated amount of essential oil is 3.0% and was obtained by hydrodistillation of dry leaves. The yield of essential oil depends on the growing conditions and the condition of the plants; from dry material, the average amount is 3.5–12.8 mL/kg (or 0.35–1.28%). The highest yield of lemongrass essential oil produced (2.63%) was obtained from dried lemongrass stems that were heated. Lemongrass can be distilled fresh or after wilting. Withering the herbs before the distillation process reduces the moisture content and increases the oil yield. Available data on the yield of essential oil obtained by this method vary significantly and range from 0.24% to 0.71% [13,30,33,34,37,38,40,41,42,43,44,45,46,47,48,49]. The oil has a scent described as lemon, even though the main ingredients of both oils—lemon and lemongrass—are different. Citral predominates in lemongrass oil (approximately 26.5% in essential oil from leaves water distillation), while limonene predominates in lemon pericarp oil (*Citrus limon* (L.) Burm.), and there is little citral [50]. However, the chemical composition of the oil (Table 1) varies depending on factors such as the geographical origin of the plant; the main ingredients are terpene hydrocarbons, alcohols, ketones, esters, and aldehydes. Among the substances isolated from lemongrass leaves and roots, the most common are alkaloids, saponins, sterols, terpenes, alcohols, ketones, flavonoids, acids, i.e., chlorogenic, caffeic, p-coumaric acids, and sugars [51]. The chemical composition of the oil may vary slightly, depending on the plant’s growth conditions, cultivation region, agrotechnics, plant age, and harvest date [13,30,33,34,37,38,40,41,42,43,44,45,46,47,48,49].

**Figure 3:**
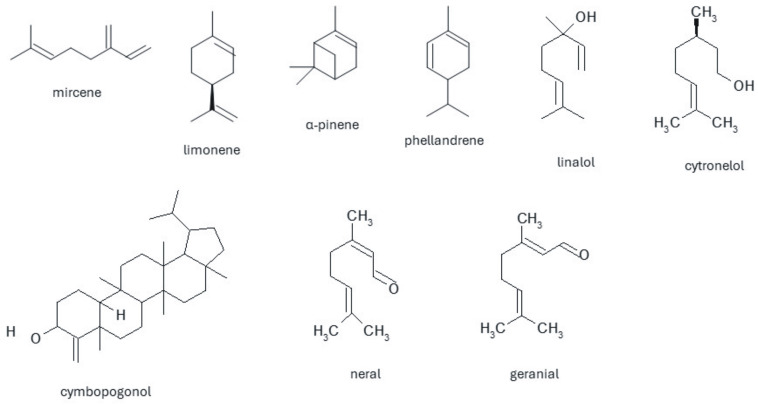
Chemical structures of selected compounds present in *Cymbopogon citratus* species extracts and essential oil.

In the group of mono-, di- and sesquiterpenes isolated from lemongrass, the most common is myrcene, which occurs in various amounts—from 2 to 25.3%, and limonene isolated in an amount of 0.2 to 13.8% [10,11,37,38,52,53,54,55]. α-pinene, α-caryophyllene, phelandrene, and α-oxobisabolene are present in smaller amounts [54]. During research on the chemical composition of wax obtained from the leaves of *C. citratus*, two chemical compounds belonging to the group of triterpenes—cymbopogon and cymbopogonol, were identified, and further research indicated that cymbopogon is probably not a natural product but an artifact produced during isolation of cimbopogonol, which in turn is classified as an alcohol. Among the ketones, ionones and methylheptenone have been isolated, constituting over 25% of the total composition of the essential oil. The main component of the oil is an aldehyde-citral (3,7-dimethyl-2,6-octadienal), which is a mixture of neral and geranial, which, depending on the place of origin of the plant, constitutes from 30 to even 93.74% of the composition [55]. This is the compound that determines the lemon scent of the plant. Research conducted in the Philippines has shown that oil obtained from plants during the dry season (March to June) contains a larger amount of citral. Hexane extraction also provides a larger amount of citral (up to 86.83%) than other solvents [55]. Isocitral, decinal and citronellal, cinnamic, salicylic, and anise aldehyde were isolated by steam distillation. The phenolic components found in lemongrass oil are primarily hydroquinone, catechol, elemycin, myrcene, quercetin, kaempferol, and apigenin [56]. The most common compound from the group of alcohols and esters is geraniol, the amount of which depends on the origin of the plant. The remaining compounds from the alcohol group are linalool, citronellol, metaheptanol, 1,8-cineol, menthol, neomenthol, terpineol, nerol, and farnesol. They are also from the group of esters: geranyl formate, citronellol acetate, terpinyl acetate, linalyl formate, linalyl acetate, and geranyl acetate. Other compounds present in the essential oil are acids-chlorogenic, caffeic, coumaric, tannins, and sitosterol (Figure 3) [46,48,57].

Extracts prepared from leaves and roots using alcohol, hexane, and chloroform differ in chemical composition. Their composition was analyzed in terms of the content of tannins, flavonoids, phenols, hydrocarbons, and essential oils, and the results obtained are presented in Table 2. Substances, such as tannins and phenolic compounds, are responsible for the antibacterial and antifungal properties of the plant. Particularly important is the content of tannins, which in herbal medicine play an important role in stopping hemorrhages because by causing precipitation of proteins, they make them resistant to the action of proteolytic enzymes. They form a membrane of coagulated protein, thus protecting the gastrointestinal mucosa [58,59,60,61,62].

**Figure 4:**
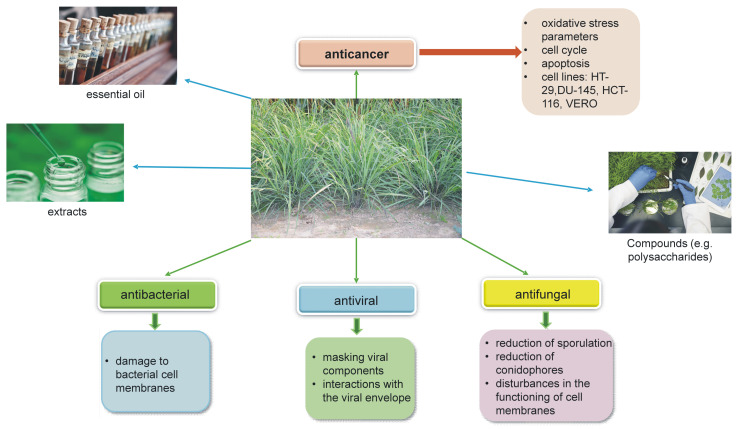
Mechanisms of biological action of essential oil and extracts from *Cymbopogon citratus*.

Both the extracts and the essential oil have antiviral, antibacterial, antifungal, and anticancer properties (Figure 4).

## 4. Antifungal Activity of *Cymbopogon citratus*

Recently, a significant increase in the number of available antifungal preparations based on plant raw materials has been observed due to the growing resistance among many species of fungi to traditionally used drugs obtained by chemical synthesis. *C. citratus* essential oil has antifungal activity against *Candida albicans*, *Candida pseudotropicalis*, *Mycosporum gypseum*, *Botritis cinerea*, *Aspergillus niger*, *Beauveria bassiana*. It is also effective against fungi from the dermatophyte group such as the following: *Trichophyton rubrum*, *Microsporum gypseum*, *Aspergillus fumigatus*, *Cladosporium trichoides*, *Trichophyton mentagrophytes*, *Epidermophyton floccosum*, *Botrytis cinerea* and *Aspergillus nidulans* [51,63,64].

The essential oil shows statistically significantly high activity against *Aspergillus niger* and *A. fumigatus* at a concentration of 5 µL/0.4 L of air. Results obtained by Sulaiman [52] also confirm the strong activity of volatile substances obtained from essential oil against the maturation of spores of both above-mentioned fungal species (Table 3). The maturation of *A. fumigatus* and *A. niger* spores was completely inhibited by volatile substances at a concentration of 10 µL/0.4 L of air, while *A. flavus* spores lost their ability to survive at a concentration of 15 µL/0.4 L of air (Table 3). Studies carried out using a light microscope showed morphological changes in the species *A. niger* after exposure to 5 µL of oil and 0.4 L of air. In control samples, a regular structure with visible hyphae containing conidia was visible. However, after exposure to *C. citratus* essential oil, the mycelium presented morphological changes, including reduced sporulation, reduced pigmentation, and the reduction and distortion of conidiophores. In addition to inhibition of hyphal growth and spore formation, disturbances in the structure and functioning of cell membranes and the disorganization of mitochondrial structures were also observed at the cellular level. Moreover, it has been shown that essential oil causes the loss of cytoplasm within the fungal hyphae, which become significantly thinner [65,66]. According to literature data, aromatic volatile compounds isolated from plants have stronger antifungal and antibacterial effects than non-aromatic compounds [67]. Essential oils are also more effective than individual ingredients isolated from them. Like essential oil, its main ingredient—citral—has antifungal activity. The action of the oil depends on the concentration of citral in it [68,69,70,71].

## 5. Antibacterial Activity of *Cymbopogon citratus*

The plant extract and essential oil have antibacterial properties. Especially the essential oil, due to the presence of citral, has a particularly strong antibacterial effect against *Staphylococcus aureus*, *Bacillus subtilis*, *Pseudomonas aeruginosa*, *Straptococcus pneumoniae*, *S. pyogenes*, *Neisseria gonorhoeae*, *Clostridium perfrigens*, *Pseudomonas fluorescens*, *Acinetobacter baumannii*, *Aeromonas veronii biogroup sober*, *Enterobacter faecalis*, *Klebsiella pneumoniae*, *Salmonella enterica* subsp. *Enetrica sorotipo typhimurium*, *Serratia marcescens*, *Proteus mirabilis*, *Shigella flexneri*, and *Salmonella typhi* [49,72,73,74,75].

The mechanism of antibacterial action of essential oil is based on the structure and properties of its bioactive substances. Hydrophobic substances such as hydrocarbons can affect chemical interactions and thus determine the structural stability of the bacterial cell and its macromolecular systems and disturb its vital functions [76]. According to Moreira et al. [77], the lipophilic components of essential oil bind to the lipid bilayer of the cell membrane, increasing its permeability and causing the cell contents to flow into the environment and damaging the cell’s enzymatic system. Souza et al. [78] found that even small changes in the structure of the cytoplasmic membrane can affect the metabolism of the bacterial cell, including the synthesis of macromolecules.

Extracts from the root and leaves of the plant prepared using various solvents (methanol, hexane, and chloroform) have various bactericidal effects (Table 4). Extracts obtained from the plant root have a stronger effect. Water and alcohol extracts from *C. citratus* have a much weaker antibacterial effect than essential oil. The water extract has no antibacterial effect at all, while the alcoholic extract has little activity. The antibacterial activity of two types of extracts and the essential oil is summarized in Table 5 and Table 6. The high antimicrobial activity of *Cymbopogon citratus* oil and extracts makes them willingly used as a replacement or supplement to synthetic preservatives used in the production of food and cosmetics [39,49,79,80,81].

## 6. Antiviral Activity of *Cymbopogon citratus*

Viral infections still remain an important problem around the world because viruses are characterized by strong resistance to both preventive measures and drugs used in antiviral therapy. Currently, only a few effective antiviral drugs are known, which is why it is so important to identify substances with antiviral properties that act not only intracellularly but also extracellularly. Methods commonly used to determine the antiviral activity of a given substance are based primarily on the inhibition of cytopathic effects, reduction in the number of viruses, and inhibition of viral functions in selected host cells in in vitro cell cultures.

There are more and more reports related to the use of plant raw materials in the treatment of viral diseases. Many essential oils have been studied for their possible use in the treatment of viral infections. In most cases, their activity against RNA and DNA-enveloped viruses was tested, i.e., herpes virus HSV1 and 2 (DNA viruses), dengue virus 2 (RNA virus), Junin virus (RNA virus), and influenza virus (RNA virus). Few essential oils have been tested for their activity against non-enveloped viruses, including oregano oil [84,85,86,87,88,89].

According to literature reports, *C. citratus* essential oil has antiviral activity against the HSV-1 virus [90]. The herpes virus causes infections that are very common in the human population, such as mucocutaneous herpes simplex infection, herpes keratitis, herpes encephalitis, and neonatal herpes. After infection, virus particles are transported through neurons to the central nervous system, where they remain in a latent state until they are activated by specific stimuli. Currently, a commonly used anti-herpes drug is acyclovir, a nucleoside analog that inhibits viral DNA replication by acting through thymidine kinase, effectively inhibiting the synthesis of viral particles. However, herpesviruses resistant to acyclovir are being isolated more and more often, which is particularly dangerous for patients with reduced immunity, e.g., HIV carriers, cancer patients, and people waiting for bone marrow or organ transplantation [91,92]. Therefore, essential oils from various plant species were tested, including *C. citratus*, as possible alternatives to traditional antiviral drugs. The best potential antiviral drugs are preparations that act on specific stages of virus biosynthesis, inhibiting specific stages of viral particle replication. Research results indicate that free viral particles are very sensitive to the action of essential oils. Herpes virus particles are inactivated just before or during adsorption to the host cell surface but not after entering the cell. Essential oils most likely act by interacting with the viral envelope or by masking viral components necessary for virus adsorption and entry into cells [84,93,94,95,96].

*C. citratus* in the form of methanol extract also shows slight inhibitory activity against the DENV-1 virus-causing dengue, which was confirmed using the MTT (the 3-(4,5-dimethylthiazol-2-yl)-2,5-diphenyl-2H-tetrazolium bromide) test [97,98,99,100,101].

## 7. Anticancer Activity of *Cymbopogon citratus*

Literature data indicate the anticancer effect of the *Cymbopogon citratus* plant, especially the essential oil and extracts obtained from this plant. According to literature data, lemongrass has anticancer effects by reducing cell viability due to increasing oxidative stress parameters in SiHa renal cancer cells. At the same time, it turns out that lemongrass has an antioxidant effect on healthy VERO kidney cells, increasing their viability and proliferation and reducing oxidative stress [102]. Lemongrass has also emerged as a promising medicinal plant that can be used as an adjunct to chemotherapy to enhance the antitumor response and reduce the resistance of prostate cancer. The studies were carried out in vitro using the DU-145 cell line. In addition to reducing the viability and proliferation of cancer cells, lemongrass also inhibits the formation of colonies. Also, in the tested cell line, it stimulates oxidative stress and causes cell cycle arrest in the G0/G1 phase. It is worth emphasizing that the extract had a selective effect on cancer cells while not causing cytotoxicity in healthy cells [103]. Ethanolic lemongrass extract has the ability to stimulate the production of reactive oxygen species (ROS) and induce apoptosis in lymphoma and leukemia cell models-MV-4-11 Chronic myelomonocytic leukemia cell line, U-937 non-Hodgkin’s histiocytic lymphoma cell line, L-540 Hodgkin lymphoma, HD-MYZ Hodgkin lymphoma, KM-H2 Hodgkin lymphoma [104]. Lemongrass extract has proven to be effective in both in vitro and in vivo studies. It induced apoptosis in colon cancer cells-colon cancer cell line HT-29 and colon cancer cell line HCT-116, depending on time and dose, while not damaging healthy cells represented in the study by normal colon mucosa cell line and normal colon mucosa cell line NCM-460 in vitro. In vivo studies showed that oral administration of lemongrass extract was well tolerated and effective in inhibiting colon cancer xenograft growth in mice. The tested extract also increased the anticancer effectiveness of drugs traditionally used in therapy and reduced their side effects, such as weight loss. Hence, it is concluded that this extract has potential as an adjunct treatment for colorectal cancer [105]. The antioxidant effect, potential anti-inflammatory effect (by inhibiting lipoxygenase), and cytotoxic effect of the essential oil of *Cymbopogon citratus* (DC.) Stapf. were also tested on various cell lines, including the prostate cancer and glioblastoma multiforme cell lines (LNCaP, PC-3, SF-767, and SF-763) [106]. Inhibition of the proliferation of oral epidermoid carcinoma cell lines under the influence of *C. citratus* essential oil has been demonstrated in mice and humans [107].

**Figure 5:**
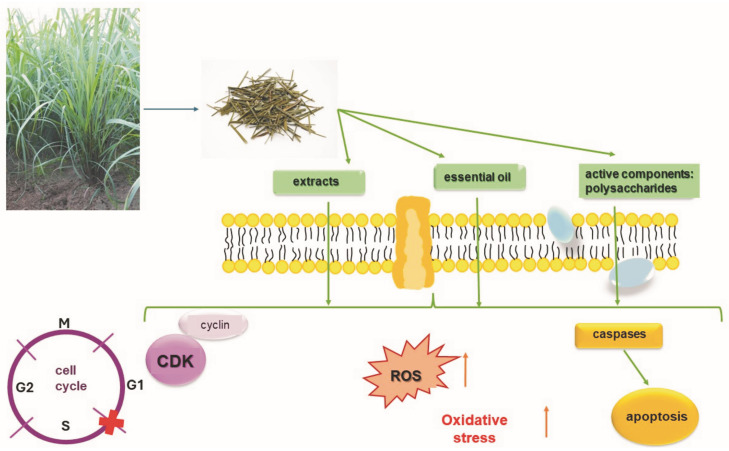
Molecular mechanisms of action of bioactive ingredients, essential oil, and *C. citratus* extracts in cancer cells. Possibility of penetration of active substances of *C. citratus* through cell membranes and induction of apoptosis in cancer cells through their influence on the activation of caspases. Increasing the level of oxidative stress in cancer cells and the effect of *C. citratus* extracts, essential oil, and selected active components on cell cycle arrest.

Several previous studies have demonstrated the anticancer effects of *C. citratus* leaf extracts, especially their bioactive components. For example, α-myrcene, α-limonene, and geraniol have been shown to have antitumor activity against breast cancer, liver cancer, and intestinal mucosa cancer cells in mouse models [108]. It is believed that *C. citratus* leaf extracts contain inhibitors of the developmental phase of skin cancer. Additionally, experiments conducted on mice confirmed that *C. citratus* leaf extract inhibits the development of colon cancer [109]. The inhibitory effect of extracts obtained from *C. citratus* on the process of hepatocarcinogenesis in rats, which was initiated by treatment with diethylnitrosamine, was also reported [110]. *C. citratus* extracts were isolated by solvent maceration, and then their antiproliferative activity was tested on five different cancer cell lines—human colon cancer (HCT-116), breast cancer (MCF-7 and MDA-MB 231), and ovarian cancer (SKOV -3 and COAV)—and a normal liver cell line (WRL 68). The obtained results suggest the antiproliferative effectiveness of the ethanol extract of *C. citratus* against selected human cancer cell lines [111]. Essential oil, which was extracted from the culm and leaf of *Cymbopogon citratus* collected from different regions of Vietnam, caused apoptosis induction and cell cycle arrest in A549 cells, as concluded based on the results obtained from flow cytometry and fluorescent nuclear staining tests. Western blot analysis showed that the apoptotic effect was caused by changes in the activity of proteins regulating the apoptosis process, such as caspase-3, Bcl-2, and Bax (Figure 5) [112].

As mentioned above, literature data indicate that essential oils obtained from *Cymbopogon citratus* have wide medical and pharmaceutical applications resulting from their numerous antibacterial, antiviral, antifungal, and anticancer properties. However, recently, there have been data indicating the biological activity of polysaccharides found in the plant and their potential anticancer effect. The anti-inflammatory and anticancer effects of *C. citratus* polysaccharides on cancer cells were analyzed in vitro. Literature data indicate that the mechanism of action of polysaccharides in inducing apoptosis in cancer cells involves inducing the internal pathway. Polysaccharide fractions had cytotoxic and pro-apoptotic effects on Siha and LNCap cancer cells. They induced apoptosis in these cells by increasing the level of caspase 3 and downregulating the bcl-2 family genes, followed by the release of cytochrome c [113]. The newly identified high molecular weight polysaccharide isolated from *C. citratus* also inhibited the proliferation of MDA-MB-231 cells, reduced the expression of cyclin D1 and CDK4, and induced cell arrest in the G0/G1 phase. This compound induced apoptosis of MDA-MB-231 cells by triggering the Fas/FasL-mediated death receptor pathway. Chen et al. indicate that the results provide a theoretical basis for the use of *C. citratus* polysaccharide as a potential anti-breast cancer agent in functional foods and medicine [114].

## 8. Limitations and Side Effects of Using *C. citratus* Products

The pharmacological use of agents derived from *C. citratus* should include restrictions on their use. Citral, which is the main component of essential oil, is considered potentially allergenic, so information about it should be clearly provided on the packaging of the dermatological preparation [115]. Machraoui et al. [9] and Ekpenyong et al. [35] also indicate possible potential toxic properties of *C. citratus* extracts at high doses. This information applies to highly concentrated preparations. However, Negrelle and Gomes [7] report that drinking a standard infusion of dried *C. citratus* does not cause any side or toxic effects. Some literature data indicate that the high content of lemongrass oil in food products may have an adverse effect on the human body, in particular on the organs of taste and smell [116]. On the other hand, lemongrass essential oil used in low concentrations is considered safe for human consumption [117]. In vitro studies have shown that the antimicrobial activity of lemongrass oil mainly ranges from 0.2 to 10 L/mL. In food products, the concentration of essential oil is much higher (25–100 times) to achieve comparable antimicrobial activity. In many countries, the use of essential oils is not regulated in any way. Moreover, improper and accidental uses of lemongrass essential oil may cause health problems due to genetic damage, carcinogenicity, and mutations [118]. Therefore, further research on the toxicity of lemongrass oil and a comprehensive safety assessment are necessary.

## 9. Conclusions

Plant medicinal preparations are becoming more and more popular in modern societies as an alternative to compounds obtained by chemical synthesis. This is due to, among other reasons, the fewer side effects they cause, the increasing availability, and the wide range of these preparations. One such raw material is lemongrass, which is very often used as a medicinal plant and in the food, cosmetic, and perfume industries. Raw materials from it are still being analyzed, and its antibacterial, antiviral, antifungal, and anticancer properties are now known. It is likely that subsequent research results will expand the possibilities of its applications in medicine. The potential anticancer properties of products obtained from lemongrass, such as essential oil and extract, as well as its chemical components, prompt extensive research on the molecular mechanisms of their action. This can be seen in the large number of emerging research results on the effects of lemongrass on cancer cells. The cytotoxic effect of this raw material on cancer cells and the cytoprotective effect on healthy cells indicate the need to continue research in this direction.

## Figures and Tables

**Table 1:** Main ingredients and their content in lemongrass leaf essential oil [10,11,37,38,46,48,49,52,53,54,55].

Main Essential Oil Ingredients	Range of Ingredient Content [%]
α-pinene	0.06–1.9
6-methyl-5-heptene-2-one	1.1–1.6
myrcen	2.0–25.3
limonene	0.2–13.8
1-8-cineol	0.12–6.4
linalool	0.1–4.82
neral ^1^	10.5–35.1
geraniol	0.4–6.6
geranial ^1^	24.9–48.0
geranylacetate	0.1–6.2
β-caryophylene	0.1–8.47
caryophyllene oxide	0.1–3.56

^1^ a mixture of neral and geranial is citral.

**Table 2:** Chemical composition of *C. citratus* leaf and root extracts [58].

Chemical Ingredients	Leaf Extract			Root Extract		
	HX	CCF	MOH	HX	CCF	MOH
tannins	-	+	+	-	+	+
flavonoids	+	+	+	+	+	-
phenols	-	+	-	-	+	-
hydrocarbons	-	+	+	-	+	+
essential oil	+	+	-	-	+	+

HX—hexane, CCF—chloroform, MOH—methanol, (-)—absence, (+)—present.

**Table 3:** Inhibitory effect of different concentrations in the air of volatile substances of lemongrass essential oil (*Cymbopogon citratus* (DC.) Stapf) on the maturation of *Aspergillus* spores.

Fungi Species	% Inhibition of Spore Maturation as Compared to the Control Sample		
	5 *	10 *	15 *
*A. niger*	83	100	100
*A. flavus*	31	68	100
*A. fumigatus*	96	100	100

Results expressed in % inhibition of spore maturation compared to the control sample [52]. * content in the air of volatile substances obtained from lemongrass (µL/0.4 L of air).

**Table 4:** Antibacterial activity of the chloroform extract of *C. citratus* [58].

Bacterial Strain	MIC (µg/mL)		MBC (µ/mL)	
	Leaf Extract	Root Extract	Leaf Extract	Root Extract
*S. aureus*	20.0	18.0	28.0	26.0
*S. typhi*	24.0	20.0	28.0	24.0
*E. coli*	14.0	14.0	16.0	16.0

MIC, Minimum Inhibitory Concentration; MBC, Minimum Bactericidal Concentration.

**Table 5:** Antibacterial activity of *C. citratus* essential oil against selected bacterial strains [82].

Bacterial Strain	Size of Microbial Growth Inhibition Zones (mm)					
	5%	10%	15%	20%	25%	30%
*Staphylococcus aureus*	14.33	19.33	22.33	24.66	27.33	29.66
*Bacillus cereus*	12.66	15.66	18.66	21.00	24.00	28.00
*Bacillus subtilis*	8.33	10.33	12.66	16.00	19.66	24.66
*Escherichia coli*	8.33	11.33	14.00	16.33	19.33	22.33
*Klebsiella pneumoniae*	7.66	9.33	11.33	12.66	14.66	17.00

**Table 6:** Antibacterial activity of alcoholic and water extracts of *C. citratus* against selected bacterial strains [83].

Medium	Size of Microbial Growth Inhibition Zones (mm)			
	*Proteus mirabilis*	*P. aeruginosa*	*S. aureus*	*K. pneumoniae*
water	0	0	0	0
ethanol	0	0	7	0

## Data Availability

Not applicable.
